# Promising roles of vitamin D receptor and APRO family proteins for the development of cancer stem cells targeted malignant tumor therapy

**DOI:** 10.32604/or.2025.059657

**Published:** 2025-04-18

**Authors:** MOEKA NAKASHIMA, NAOKO SUGA, AKARI FUKUMOTO, SAYURI YOSHIKAWA, SATORU MATSUDA

**Affiliations:** Department of Food Science and Nutrition, Nara Women’s University, Kita-Uoya Nishimachi, Nara, 630-8506, Japan

**Keywords:** Vitamin D, Cancer stem cell, Invasion, Metastasis, Chemoresistance, Ferroptosis, Tumor microenvironment, Noncoding RNAs, Cancer therapy

## Abstract

Malignant tumors are heterogeneous diseases characterized by uncontrolled cell proliferation, invasion, metastasis, and/or recurrence of their malignancies. In particular, cancer stem cells (CSCs) within these tumors might be responsible for the property of invasiveness and/or therapies-resistance. CSCs are a self-renewing, awfully tumorigenic subpopulation of cancer cells, which are notorious for strong chemoresistance and are frequently responsible the aggravated invasion, metastasis, and/or recurrence. Developing targeting therapies against CSCs, therefore, may be deliberated a more encouraging mission for the greater cancer therapy. Innovation for a more potent anti-CSC treatment has been required as soon as possible. Interestingly, vitamin D could modulate the inflammatory condition of the tumor microenvironment (TME) by successfully affecting CSCs, which has an imperative role in determining the malignant phenotype of CSCs. In addition, vitamin D may also contribute to the regulation of the malignant behaviors of CSCs. Consistently, vitamin D could have potential applications for the significant inhibition of several tumor growths within various cancer therapies. The biological significance of vitamin D for CSCs regulation may be involved in the function of APRO family proteins. Therefore, vitamin D could be one of the innovative therapeutic modalities for the development of novel CSCs related tumor therapies.

## Introduction

Malignant tumors may be considered by high mortality rates. In addition, cancer death may be the top cause of human death. Therefore, malignant tumors are an enormous threat to humans, which is also an important public health concern. Some clues of therapeutic intervention against this intractable disease should be obtained. Now, a theory of cancer stem cells (CSCs) has deep attention in researching this malignant tumor therapy. CSCs are a small population of cancer cells possessing the quality of cell-stemness like ordinary stem cells. They can achieve self-renewal to yield daughter cells with cell-stemness and/or usual cancer cells without cell-stemness [1]. The self-renewal capacity may exhibit increased fraction during the tumor progression. In general, the CSCs are holding special tumor microenvironment (TME), which may render high tumorigenic potential to CSCs [2]. The TME may include tumor cells themselves and tumor-associated cells, which could together support their effector functions. The concept of CSCs may indicate that some tumors can easily become resistant to several cancer-therapies, which may also suggest the recurrence of malignant tumors [3,4]. Their capacity to evade cancer-therapies makes them a focus of intense research as for the development of potential therapeutic applications [5]. In particular, investigations have revealed a close association between the development of CSCs and the TME for their malignancy levels [6]. Therefore, alterations in the TME may exert insightful effects not only on the CSCs-response within tumors but also on the efficacy of cancer-therapies [7].

CSCs can intensify tumorigenic potential underlining the aggressive character of tumor cells [8]. In addition, some genetic mutations and epigenetic modifications can also lead to the reprogramming of the CSCs, endowing them with more stem cell-like properties. Various factors are thought to be critical in the alteration of CSCs as well as the TME, which could become more risky to the host [9,10]. The intricate molecular mechanisms in their signaling pathway for the regulation of CSCs may also be required depending on various tumor types. It has been shown that some transcription factors including SOX2, OCT4, KLF4, and/or Nanog may achieve the regulatory effects on the pluripotent condition of CSCs [11]. Concurrently, additional signaling pathways such as PI3K/AKT/mTOR, TGF/SMAD, JAK/STAT, PPAR, and Wnt may also play crucial roles in the signaling network within CSCs [12]. Interestingly, vitamin D has been shown to control inflammatory conditions of the TME by affecting CSCs [13]. In addition, vitamin D can increase the efficacy of anticancer drugs such as cisplatin, doxorubicin, gemcitabine and as well as proton cancer therapy [14–16]. Along with the positive antitumor efficacy, vitamin D supplementation could satisfactorily reverse the chemotherapy resistance [17,18]. In agreement with this, vitamin D deficiency may promote resistance to chemotherapies [19]. Furthermore, vitamin D could reverse the multidrug resistance to the cancer therapy [20,21]. Interestingly, vitamin D could reduce the expression of stem cell markers such as SOX2, OCT4, KLF4, and/or Nanog in stem-like cells, which can decrease the sphere formation [22,23]. Consistently, decreased vitamin D receptor (VDR) expression has also been observed to be associated with impaired myeloid progenitor cell differentiation in acute myelogenous leukemia (AML) [24]. In this case, the suppression of VDR may mostly be by reason of gene promoter methylation, which can promote self-renewal and/or proliferation in myeloid stem cells. Accordingly, VDR activation by several vitamin D agonists may inhibit cell stemness in AML cells [24]. Consequently, vitamin D could be a therapeutic modality for the development of CSCs related tumor therapies. Here, this research direction has been discussed for the innovative cancer treatment strategy probably associated with certain benefits for several tumor patients.

## VDR Signaling May Be Associated with the Efficacy of Cancer Therapies

**Figure 1:**
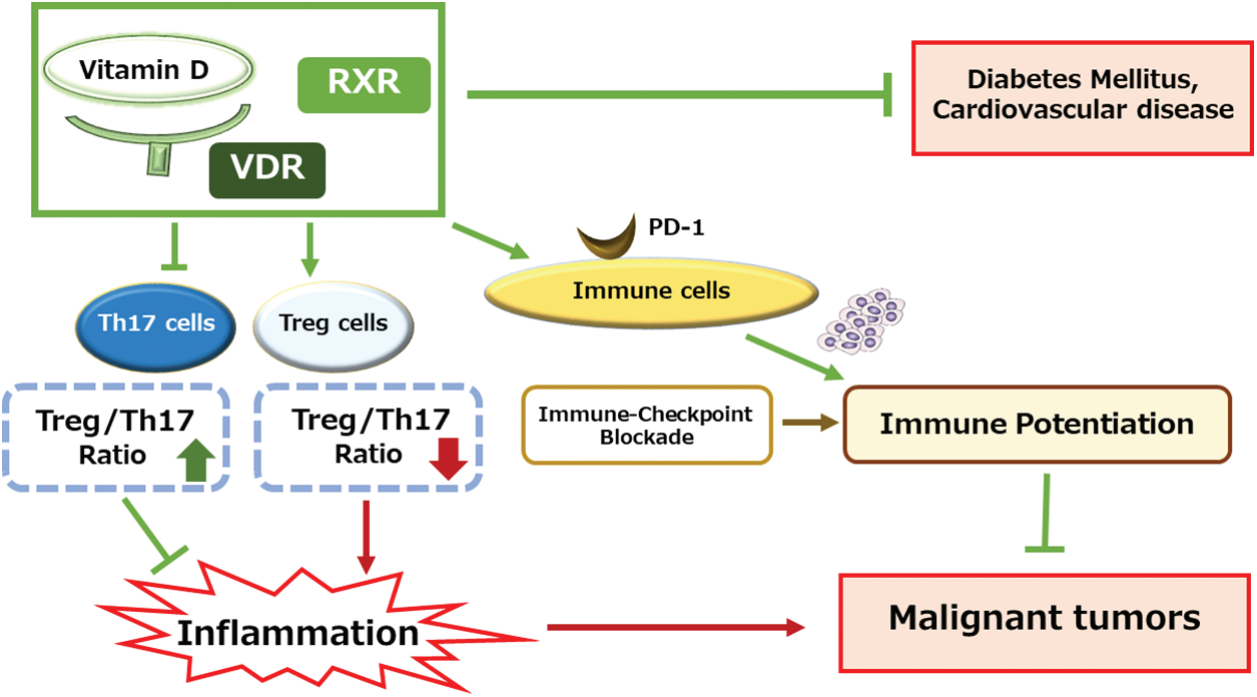
Illustration of the relationship between vitamin D signaling and several diseases. Through the association of vitamin D, its receptor (VDR) and retinoid X receptor (RXR), the signaling could protect the host from several diseases including malignant tumors. In particular, adjusted Treg/Th17 cells balance may contribute to the inhibition of inflammation and/or potentiation of immune cells, which might prevent the development of malignant tumors. Arrowhead indicates stimulation whereas hammerhead shows inhibition. Note that several important mechanisms for the protection of each disease such as redox-modification and/or anti-inflammatory pathway have been omitted for clarity. Abbreviation: VDR, vitamin D receptor; RXR, retinoid X receptor; ncRNAs: noncoding RNAs; ROS, reactive oxygen species; PD-1, programmed cell death protein 1; TME, tumor microenvironment.

The active form of vitamin D is principally known as a crucial regulator of calcium and/or phosphate homeostasis, which can exert these functions by attaching to the VDR, a kind of transcription factor that regulates various gene expression in several organs such as bone, intestine, and kidney. In general, vitamin D could protect from the poor health outcomes of the host such as heart disease, diabetes, and/or cancers [25,26] (Fig. 1). Therefore, biological properties of vitamin D may include cellular protection by regulating the cellular proliferation and/or immune function as well as cell differentiation and/or cell death/apoptosis [27]. These mechanisms could be additionally controlled in conjunction with retinoid X receptor alpha (RXRa) [28]. The VDR-RXRa complex can bind to vitamin D response elements that can also stimulate and/or suppress several gene transcription [29]. Biologically, the VDR is expressed in several normal and cancerous tissues, where it can regulate the proliferative, differentiating, and/or immune-modulating effects. In addition, it has been revealed that the VDR could impact on the population of CSCs indicating novel insights on the implications for the application of vitamin D in several cancer therapies [30]. An epidemiological study has shown that low levels of vitamin D are associated with an increased risk of breast cancer development [31]. Remarkably, it is well-known that the resistance of breast cancer to tamoxifen is mediated by the CSCs [32]. Interestingly, vitamin D has been considered to have probable applications for the inhibition of tumor growth in cancer therapy [33]. For example, a ligand for VDR, calcipotriol, can decrease the levels of inflammation in the pancreas cancer tissue, suggesting that VDR could satisfactorily reduce tumor volumes with improved survival rates [34]. In addition, administration of vitamin D may activate the VDR of host normal cells, which can concomitantly inhibit the Wnt/β-catenin signaling in cancer cells with increased sensitivity to chemotherapies [19,35]. Furthermore, epidemiological studies have suggested an association between vitamin D deficiency and inflammatory bowel diseases as well as colorectal cancers [36,37]. Similarly, cell lines and animal models of colorectal cancer have shown a protective role of VDR agonists [38,39]. In addition, the regulatory role of vitamin D on the colon CSCs has also been reported [40]. The association of VDR agonists can promote the exhaustion of leukemia stem cells in AML mouse models, which may indicate a novel therapeutic direction immediately applicable to AML patients [24,41]. Interestingly, the damage of radiation-induced intestinal stem cells can be reduced by VDR activation [42]. Consistently, upregulation of VDR expression can mitigate radiation-induced intestinal damage, in which enhanced VDR promotes the repair of epithelial damages [42]. On the contrary, loss of VDR expression in the intestinal stem cells can lead to considerable damages to stem cells, which may be associated with restricted Wnt/β-catenin signaling in stem cells [43]. In addition, vitamin D deficiency could also initiate cytokine dysregulation and/or immune imbalance, which may contribute to several autoimmune diseases [44]. Consequently, the expression of VDR might indirectly be related to the initiation and/or the development of tumors. However, the regulatory mechanism of VDR expression in CSCs has been still unclear. Clarification of the effect of vitamin D and/or VDR on the stemness in CSCs might be indeed indispensable [45]. In addition, elucidation of the interaction between CSCs and the TMEs in the presence and/or absence of vitamin D might also be required.

## A Microenvironment for Ferroptosis of Cancer Stem Cells May Be Related to the VDR Signaling within Tumor Cells

**Figure 2:**
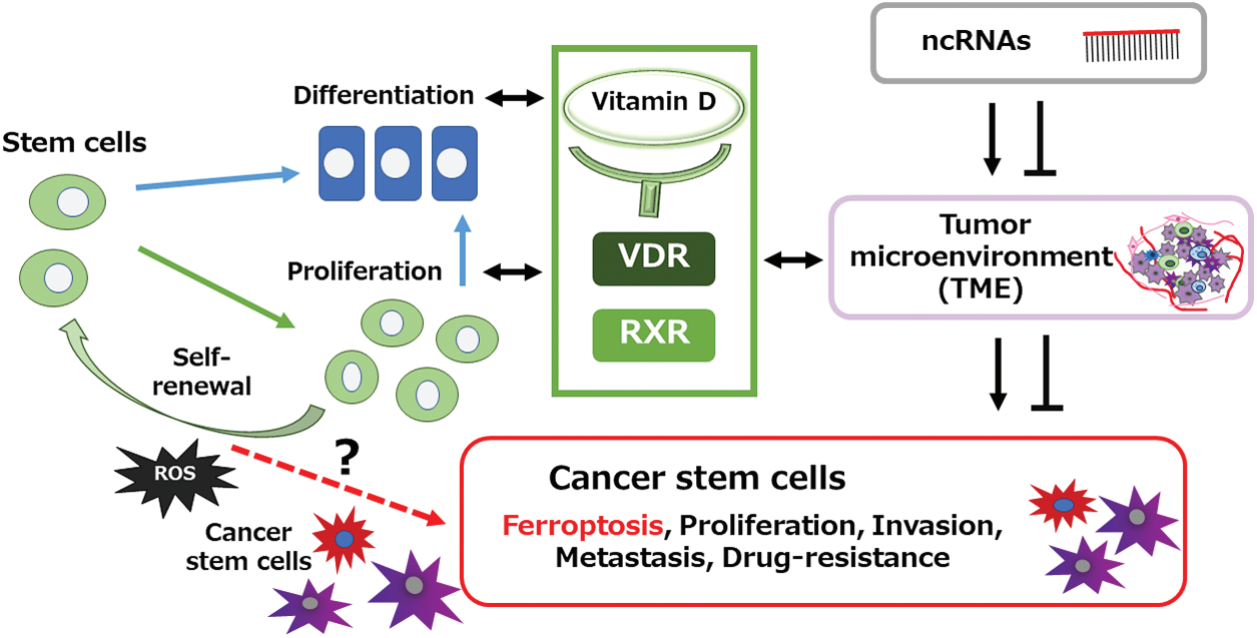
An image of the relationship between vitamin D signaling and some characters of cancer stem cells. Cancer stem cells and/or normal stem cells can keep the population of these stem cells through appropriate self-renewal and proliferation activities. Surrounding tumor microenvironment (TME) with the regulation of ncRNAs might also be involved in the homeostasis via the acquisition and/or maintenance for features of cancer stem cells. Some levels of reactive oxygen species (ROS) may prime cancer stem cell-formation conveyed from normal stem cells. Arrowhead means stimulation, whereas hammerhead represents inhibition. Note that some critical pathways have been omitted for clarity. Abbreviation: VDR, vitamin D receptor; RXR, retinoid X receptor; ncRNAs: noncoding RNAs; ROS, reactive oxygen species; TME, tumor microenvironment.

TME has an important role in determining the phenotype of CSCs, which may also contribute to the behaviors of CSCs [46]. It is well-known that low oxygen and/or low pH conditions are two characteristics of the TME, which may lead to a series of alterations related to an induced phenotype and/or a reprogramming phenotype of CSCs [47]. Appropriate places for surviving stem cells may consist of a beneficial microenvironment of adjacent cells and/or surrounding extracellular matrix, which can provide special signals that could protect the stem cells [48]. Increasing evidence has shown that the limited availability of amino acids in the TME could make the functionality of CSCs, while also inhibiting uncontrolled proliferation of CSCs [49]. Interestingly, cysteine may be the limited amino acid in the TME due to its high consumption by both tumor cells and immune cells [50]. It is well-known that cystine and cysteine are indispensable for the cell proliferation and/or the protein/DNA synthesis [51]. In addition, cystine can protect cells from oxidative damages by its anti-oxidative properties in the TME [52]. Cystine could easily be reduced to cysteine, which is the rate-limiting amino acid for glutathione synthesis to maintain the redox homeostasis in cells [52]. Remarkably, the cystine deprivation can induce ferroptosis in tumor cells [53]. Oxidative stress, Fe^2+^, and lipid accumulation are key factors that contribute to ferroptosis. In addition, cystine deprivation may also cause the augmented peroxidation of lipids [54]. Furthermore, cystine deprivation could repress the clearance of lipid peroxides [55]. Notably, cystine deficiency-induced glutamate accumulation has been shown as a pivotal manager of reactive oxygen species (ROS) production, which is closely associated with ferroptosis in various tumor cells [56] (Fig. 2).

In these ways, ferroptosis, a form of non-apoptotic cell death, is dependent on the accumulation of ROS in various cells [57]. The ROS production and aberrant iron metabolisms are some of the physiological differences between cancer cells and normal cells [58]. Since these characteristics may play essential roles in regulating the growth of tumors, CSCs could be more susceptible to the modulation of ferroptosis pathway compared to normal cells [59]. In addition, ferroptosis inducers may obviously target the CSCs in tumor, in which CSCs could definitely be predisposed to ferroptosis [60,61]. Therefore, ferroptosis may play an imperative role in tumors therapy, as ferroptosis could possess massive efficacy in inducing the death of CSCs [60]. Interestingly, it has been shown that the suitable activation of VDR is protective against cisplatin-induced acute kidney injury (AKI) through preventing ferroptosis [62]. A few studies have also revealed that the activation of VDR can also impede the ferroptosis of osteoblasts by activating the Nrf2 signaling pathway [63]. Additionally, activated VDR can also hamper the ferroptosis in zebrafish cells by controlling the Nrf2 and NF-κB signaling axis [64]. Moreover, the VDR can activate the Nrf2/HO-1 signaling pathway thus declining the ferroptosis also in aging mice [65]. Therefore, vitamin D could efficiently control the ferroptosis within CSCs. Inhibiting the Nrf2 signaling pathway might decrease the protective effect of vitamin D against ferroptosis (Fig. 2).

## Vitamin D, Autophagy and Ferroptosis against the Pathology of Tumors with the Alteration of Gut Microbiota

Remarkably, vitamin D could induce autophagy, which plays an important role in both cell survival and cell death [66,67]. Vitamin D could also change the mode of autophagy from survival to death in cancer cells [68]. It has been shown that calcitriol-stimulated autophagy may play a critical role in the regulation of both autophagy and cell death [69]. Additionally, vitamin D-induced autophagy is a consequence of the DNA damage response 1 protein expression, which is known as an inhibitor of the mechanistic target of rapamycin complex 1 (mTORC1) that suppresses autophagy [70]. Although autophagy keeps optimal cellular homeostasis under the physiological conditions for cell survival, excessive autophagy could result in inducing autophagic cell death [71]. Autophagy may contribute to cell survival as well as to cell death [71]. Whether cell survival or cell death might probably depend on the damage level in a cell. Again, CSCs could be more susceptible to the DNA damage-response pathway compared to normal stem cells [59]. Evidence indicates that an intricate crosstalk might exist between the autophagy and apoptotic programmed cell death [72], in which excessive activation of autophagy can increase ferroptosis in tumor cells [73]. Therefore, autophagy, apoptosis and/or ferroptosis may be showing some modes of cell death implicated in the anti-tumors effect of several cancer chemotherapies [74]. Interestingly, it has been shown that some types of cell death including apoptosis, autophagy and/or ferroptosis may also provide a significant link to the cross-talk of the host gut microbiota [75]. The gut microbiota might be an environmental factor for the host, in which several cell/tissue/organ interactions may play key roles in making them susceptible or resistant to carcinogenesis. In fact, a noteworthy relationship between tumor development and the alteration of gut microbiota has been suggested [76]. In addition, some microbiomes in the gut might help to prevent the development of tumors as well as the positive responses to cancer chemotherapies [76].

The gut microbiota can produce some beneficial metabolites to the host such as short-chain fatty acids (SCFAs), which could influence the progression of several diseases including cancers [77]. The SCFAs can also affect immune responses against cancers [78]. In general, the SCFAs mainly consist of acetate, propionate, and butyrate, which could promote the differentiation and/or the proliferation of immune cells [79]. In particular, butyrate can promote anti-tumor immunity by promoting CD8^+^ T cells to play a key role for the inhibition of tumors in TME [80]. Moreover, butyrate possesses a robust cell proliferation-inhibitory effect on cancer cells [81]. Butyrate can also enhance the novel immunotherapy against various tumors by boosting the CD8^+^ T cells [82]. Furthermore, butyrate can significantly induce the apoptosis of cancer cells [83]. Therefore, butyrate could present several beneficial anti-cancer effects [84]. Interestingly, it has been shown that butyrate may enhance ferroptosis through modulating the antioxidant Nrf2 signal pathway [85]. In addition, butyrate could also alleviate the ferroptosis via the AMPK signaling to induce autophagy and/or Nrf2 signaling responses [86]. The treatment with butyrate could particularly decrease ferroptotic resistance of CSCs [87]. Mechanistically, the butyrate can activate a G-protein-coupled receptor within cancer cells, which could assist the autophagy of radiotherapy against colorectal tumors [88]. In addition, the histone modification induced by butyrate may epigenetically be linked to the activation of several gene expression contributing to both apoptosis and inhibition of cell cycle [89]. Remarkably, it has been shown that butyrate can significantly control the number of CSCs using the transcription factors SOX2, OCT4, KLF4, and c-Myc possibly via the modification of epigenetic mechanisms [90].

## Possible Treatment Tactics against Tumors with CSCs

Active vitamin D and some VDR agonists could exert anticancer effects including cell differentiation and/or apoptosis against invasion/metastasis of VDR-positive tumor cells [91]. Furthermore, vitamin D could increase the effectiveness of anticancer drugs in several models of tumor [16,92]. In addition to the synergistic antitumor efficacy induced by vitamin D supplementation, an evolving role of vitamin D as a reversal agent for the multidrug drug resistance induced by several chemotherapies has been increasingly described [19,93,94]. Now, the VDR could play an imperative role in the drugs interaction for retrieving more effective and/or safer cancer treatments [93]. Consistently, low levels of circulating vitamin D are associated with an increased risk of tumors, whereas supplementation of vitamin D in combination with other immunotherapeutic drugs may improve therapeutic consequences [95]. For example, VDR activators can diminish the cisplatin-induced ferroptosis in kidneys by reducing lipid peroxidation [62,96]. The VDR activation can also diminish the androgen-induced ferroptosis of granulosa-like tumor cells [96]. However, vitamin D can promote ferroptosis in CSCs, which may influence the expression of TP53 tumor suppressor protein [97]. In addition, induction of the ferroptosis has become a promising cancer therapy in cancer patients, particularly for malignant tumors that are resistant to conventional chemotherapies [98,99]. Again, it has been identified that the relationship between the VDR and gut microbiota could play important roles in the regulation of autophagy and/or ferroptosis beneficial for malignant tumors treatment [100]. Supplementation of vitamin D might also contribute to affect the alteration of gut microbiota, which could facilitate the effect of vitamin D [101]. Indirect evidence of a potential interaction between vitamin D and gut microbiota has been presented by several findings, which has recognized a synergistic effect of vitamin D and some gut metabolites such as butyrate in enhancing the expression of phosphatase and tensin homolog (PTEN), a tumor suppressor protein, and resultant tumor cells death [102,103]. This synergistic effect of butyrate have also been observed for the cancer cell differentiation/apoptosis in the colorectal cancer cell line cells (Caco-2) [104]. In addition, butyrate could influence the immune response against tumor cells via indorsing T cell differentiation into effector T cells [105]. Therefore, butyrate concentrations may be correlated with therapeutic outcomes of malignant tumor patients. Interestingly, the radio-sensitivity may enhance in patients with lack of vitamin D, which can be decreased by the activation of the VDR [106]. Ensuing certain VDR activation, therefore, the radiation-induced damages of CSCs may be reduced moreover [42,106]. In other words, the VDR could protect against radiation-induced damages through inhibition of stem cell apoptosis in clinical radiation therapy [106].

Members of the anti-proliferative (APRO) protein family such as transducer of ErbB-2 (Tob)-1 and Tob-2 may be involved in the proliferation of mouse embryonic stem cells, however, which may not compromise the fundamental properties of CSCs [107]. Interestingly, it has been shown that the adenovirus-mediated *tob1* expression has potential to suppress the formation of pancreatic cancer peritonitis, which could be applied for chemotherapy-resistant cancer peritonitis [108]. The expression of APRO family members may be linked to drug sensitivity as well as the prognosis of cancer patients. In addition, APRO family genes have shown significant association with the TME, tumor cell stemness, and immune infiltration subtypes [109]. Furthermore, some APRO family members may also be involved in the regulation of cancer cell migration and/or invasion [110]. The human APRO family may include six members, namely Tob-1, Tob-2, B cell translocation gene 1 (BTG1), BTG2/tetradecanoyl phorbol acetate-inducible sequences 21 (TIS21)/pheochromocytoma cell-3 (PC3), BTG3/abundant in neuroepithelium area (ANA) and BTG4/PC3B, which may all play important roles in regulating cell proliferation, invasion and/or apoptosis of various cancer cells [111]. In addition, it has been reported the role of APRO family proteins may be involved in the regulation of immune responses by Th17 cells. For example, Tob-1 can decline the IL-2 production in the Th17 cells, and Tob-1 can also impede the expression of several cell cycle-related genes, suggesting that Tob-1 might suppress the proliferation of Th17 cells during some pathways including signal transduction, RNA transcription, and/or post-transcriptional regulation of RNAs [112]. The biological function/mechanism of APRO family proteins might be quite complicated, however, which may be a crucial modulator of non-coding RNAs (ncRNAs) such as microRNAs (miRNAs) [113].

**Figure 3:**
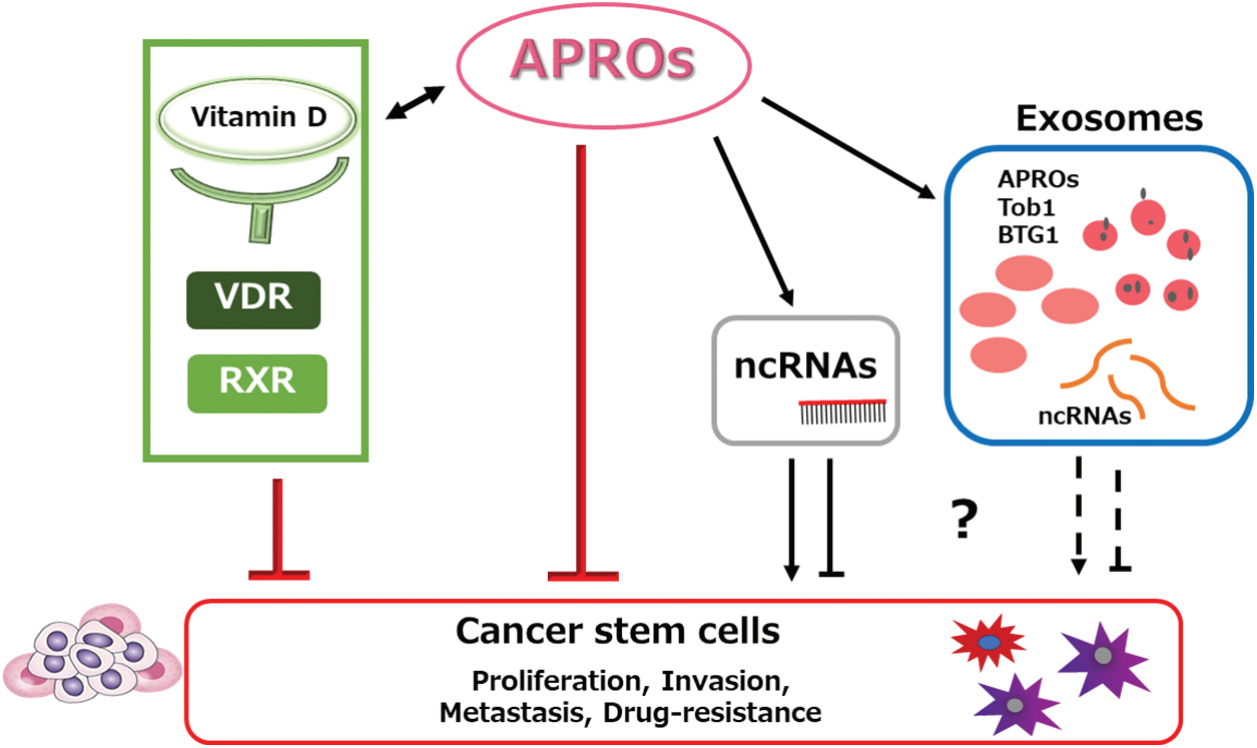
Hypothetical schematic image of the relationship among APRO proteins (APROs), noncoding RNAs (ncRNAs), exosomes, and the cancer stem cells has been shown. In addition to the individual APROs’ potential for the suppression of tumor progression, invasion, metastasis, and/or probable drug-resistance by cancer stem cells, the presence of some exosomes and/or noncoding RNAs (ncRNAs) could further alter the feature of cancer stem cells. Indicated molecules are shown as some examples. Arrowhead means stimulation whereas hammerhead represents inhibition. Note that some critical pathways such as the regulation of relevant immune cells have been omitted for clarity. Abbreviation: APROs, APRO family proteins; ncRNAs, noncoding RNAs; VDR, vitamin D receptor; RXR, retinoid X receptor.

One member of the APRO family may interact with the VDR [114]. For example, a lower concentration of the VDR mRNA may be observed at the transcriptional level, together with the upregulation of the *tob2* gene in the *APRO* family genes [115]. In addition, vitamin D may induce the differentiation of HL-60 human promyelocytic leukemia cells partly via the upregulation of BTG1, another member of APRO family proteins [116]. Furthermore, the VDR signaling may also contribute to the upregulation of BTG2, the other member of APRO family proteins, via the expression of miR-1278 [117]. Therefore, the biological significance of vitamin D may be partly involved in the regulation of APRO family proteins. As the key role of the APRO family proteins may be involved in the regulation of ncRNAs function [118], it is possible that vitamin D might employ the action of APRO family proteins for the relevant CSCs regulation (Fig. 3).

## Conlcusions and Forthcoming Perspectives

Some ncRNAs might be potent regulators of tumorigenesis in various cancers. For example, the abnormal expression of several miRNAs can be detected in certain malignant tumor cells [119]. Enrichment of the specific miRNAs can be also observed in exosomes to further clarify their application as consistent diagnostic and/or prognostic modalities [119]. Evidence has shown that several miRNAs enclosed in exosomes could be sheltered from immune attacks. Those ncRNA’s interactions with CSCs might facilitate tumor development, invasion, metastasis, and/or therapy resistance, which could represent the development of superior cancer therapeutic tactics. In fact, current studies have emphasized the significance of ncRNAs in CSCs-induced chemoresistance [120]. Several studies have also shown that the crosstalk between CSCs, ncRNAs, and the TME could support the proliferation, survival, stemness, and invasion of CSCs [121]. In addition, CSCs have been shown to develop neovascularization by expressing various angiogenic factors via the utilization of various ncRNAs, which could contribute to their own maintenance of CSCs [122]. Actual progression of tumors might critically rely on the function of some mechanisms of CSCs including the neovascularization and/or the adjustment of TME with the rigorous ncRNAs interactions.

As for APRO family proteins, the mechanism of anti-proliferative property against CSCs is still unclear. Possibly, the Treg immune cells may play a vital role in the TME of CSCs, in which Tob-1 could exhibit close associations with infiltrating Treg cells within several malignant tumors [123]. Gathering evidence has revealed that the aberrant expression of ncRNAs can play a significant role in the pathogenesis of several diseases including malignant tumors. Remarkably, certain ncRNAs and several RNA-binding proteins including the APRO family proteins could be intensely linked to the incidence and/or maintenance of malignant tumors [124]. Therefore, it is imperative that the biological impact of vitamin D could be involved in the regulation of APRO family proteins. Interestingly, treatment with vitamin D can significantly induce ferroptosis in colorectal CSCs, suggesting a therapeutic use of vitamin D in treating various malignant tumors [125]. In addition, vitamin D signaling could contribute to the differentiation of Treg cells, which could inhibit tumorigenesis possibly via the modulation of immune checkpoint molecules [126]. Based on the other clinical trials to bone disease, nephrologists are cautious to administer high doses of vitamin D to dialysis patients for fear of vitamin D toxicity characterized by hypercalcemia and/or hyperphosphatemia [127]. However, the correction of vitamin D insufficiency in patients with chronic kidney disease could be safely and effectively accomplished with one weekly dose of 200,000 IU oral cholecalciferol for 3 weeks without obvious toxicity [127]. Therefore, tumor -treatments with vitamin D could be one of the innovative therapeutic modality for the enhanced success of immune checkpoint cancer therapies. Further investigations are indispensable to fully understand the molecular mechanisms for the promising clinical consequences as innovative therapeutic strategies.

## Data Availability

Not applicable.

## Abbreviations

AKI: Acute kidney injury
AML: Acute myelogenous leukemia
ANA: Abundant in neuroepithelium area
APRO: Anti-proliferative
BTG1: B cell translocation gene 1
CSCs: Cancer stem cells
cAMP: Cyclic adenosine monophosphate
ECM: Extra-cellular matrix
MAPK: Mitogen activated protein kinase
miRNAs: MicroRNAs
ncRNAs: Noncoding RNAs
mTORC1: Mechanistic target of rapamycin complex 1
PC3: Pheochromocytoma cell-3
PD-L1: Programmed cell death ligand 1
PD-1: Programmed cell death protein 1
PTEN: Phosphatase and tensin homolog
RXR: Retinoid X receptor
RXRa: Retinoid X receptor alpha
ROS: Reactive oxygen species
SCFAs: Short-chain fatty acids
TIS21: Tetradecanoyl phorbol acetate-inducible sequences 21
TME: Tumor microenvironment
Tob: Transducer of ErbB-2
VDR: Vitamin D receptor
